# Case of Carcinoma of the Eyelid

**Published:** 1853-01

**Authors:** C. Theodor Meier

**Affiliations:** New York.


### ARTICLE XV.

*Case of Carcinoma of the Eyelid—Blepharoplastic Operation after Dieffenbach's Method.* By C. THEODOR MEIER, M. D., of New York.

MRS. L., sixty-eight years old, with the exception of some derangement of digestion caused by hypertrophy of the liver, enjoyed, in general, good health, noticed about ten years ago a wartlike excrescence upon the lower eyelid of the left eye, which, growing slowly, was removed four years ago by simple excision. But again a hard swelling in the eyelid and upon the lower margin of the orbit developed itself gradually in size, and encroached upon the conjunctiva, the orbit, and downward upon the cheek. When I saw the patient in October, 1851, the hard tumor with an uneven surface was very little movable, therefore, I anticipated the tumor being adherent to the periosteum. In November the skin of the eyelid began to ulcerate; and in January, 1852, a deep excavation, with hard thick edges showed itself, from which a serous and bloody liquid was discharged. The diagnosis as of carcinoma of the eyelid was clear enough. The patient suffered a great deal from lancinating pains, hemorrhage, inflammation of the conjunctiva, and want of sleep; and having had the advice and different opinions of professional gentlemen about the disease, desired the operation very strongly.

On the 18th of February, 1852, the operation was performed with the kind assistance of Dr. Tellkampf and Dr. Gurdon Buck, of New York. No ether or chloroform was administered, owing to valvular disease of the heart.

From the interior corner of the eyelid next to the lachrymal point, I commenced an incision downward to the cheek, then from the same point close to the border of the eyelid a horizontal incision was made to the external corner, thus preserving a very small linear part of the border of the eyelid. A third incision, beginning at the termination of the second, was carried downward to the cheek, connecting with the first in an acute angle. These three incisions formed a triangular space, and included all the diseased parts, which were removed as carefully as possible, being in intimate connection with the periosteum, and stretching into the orbit and partially under the eyeball itself.

This great defect of the eyelid had now to be replaced, and this was done in the following way: A nearly horizontal incision, from the external corner of the eyelid to the temple, about one inch and three quarters long, was made, and from the termination of this incision the scalpel was carried downward over the cheek nearly parallel with the external side of the triangle that was formed by the defect; the incision terminated on the same line with the inferior point of the triangle. Both these incisions included the flap designed for the new eyelid, and then a thick flap was separated from the subjacent tissues. After the bleeding stopped, the flap was sidewards displaced and adapted to the defect of the eyelid, so that the above mentioned triangular space was completely covered. The inner border of the flap was now brought in connection with the inner side of the triangle by means of six interrupted sutures; the horizontal border of the flap was united with that very small remaining part of the border of the eyelid by four sutures; besides these, one suture was applied on the external commissure of the eyelid. The deficiency of skin over the cheek, wherefrom the flap was taken, was left ununited only with the exception of a single suture on the most external part of the wound.

The raw surface of the wound over the cheek was covered with lint moistened with warm water, and ice-water applications ordered. The night of the following day, high fever, inflammation, and swelling of both eyelids, set in, extending even to the right eye. Leeches were applied behind the left ear, and ice-water continually applied during two days and nights over the face and forehead. From the fourth to the ninth day, all the pins and sutures were removed one after the other, and a few small straps applied. The flap had united by its borders to the surrounding parts, but the posterior surface of the flap did not unite immediately with the subjacent tissues; and in the first weeks a great deal of purulent matter was discharged, that forced its way partially through the newly-united inner corner of the eyelid, partially through the outer border of the flap. This discharge ceased after the lapse of seven weeks, the flap being entirely adherent at all parts; the wound over the cheek had, three weeks after the operatien, completely cicatrized, and left a very small scar. The shape of the eyelid appeared very good, only a slight ectropium had gradually formed in consequence of the contraction of the transplanted eyelid. As the carcinomatous destruction had involved most of the fibres of the orbicular muscle, the mobility of the eyelid was limited. The first week after the operation a prick with the point of a needle on the upper part of the flap could not be felt; but in the second week normal sensation recurred. Up to this time, July, 1852, no relapse has taken place, and the general health of the patient is very satisfactory.

The method on which this operation was performed was Dieffenbach's method of sideways displacement, *(déplacement lateral,)* a method he improvised at first in 1835, at Paris, in Lisfranc's Clinique, on a patient, whose inferior eyelid was destroyed by carcinoma. The idea of this method is, that the wound over the cheek, healing by the gradual process of granulation and cicatrization, stretches and draws out the surrounding tissues, especially the flap itself, thereby preventing the new eyelid from contracting or coiling upon itself, as is always the tendency with transplanted skin. Left to this tendency, the object of forming a natural-looking eyelid could not be attained, and instead of the regular shape of the eyelid, there would be seen in its place a round and prominent piece of skin.

The second advantage which this operation possesses is, that the flap, having a broad root, keeps up a free and undisturbed connection with the surrounding parts, from which the nourishing vessels of the flap enter; and the root of the flap, being formed close to the defect, is not exposed to be turned and twisted, as would be the case, when the flap is taken from a more remote part, were the connecting root has to be turned about two right angles. With this proceeding there is greater probability that the circulation of the flap will not be disturbed, and the chance of preserving the flap, and of union by the first intention, is therefore greatly increased.—*N. Y. Jour. Med.*

